# Emergence of Melioidosis in Indonesia and Today’s Challenges

**DOI:** 10.3390/tropicalmed3010032

**Published:** 2018-03-13

**Authors:** Patricia M. Tauran, Sri Wahyunie, Farahanna Saad, Andaru Dahesihdewi, Mahrany Graciella, Munawir Muhammad, Delly Chipta Lestari, Aryati Aryati, Ida Parwati, Tonny Loho, Dewi Indah Noviana Pratiwi, Vivi Keumala Mutiawati, Ricke Loesnihari, Dewi Anggraini, Siwipeni Irmawanti Rahayu, Wahyu Nawang Wulan, Ungke Antonjaya, David A. B. Dance, Bart J. Currie, Direk Limmathuthurotsakul, Mansyur Arif, Abu Tholib Aman, Ni Nyoman Sri Budayanti, Diah Iskandriati

**Affiliations:** 1 Department of Clinical Pathology, Faculty of Medicine, Universitas Hasanuddin/Dr. Wahidin Sudirohusodo Hospital, Makassar 90245, Indonesia; mansyurarif64@gmail.com; 2 Indonesia Research Partnership on Infectious Diseases (INA-RESPOND), Jakarta 10560, Indonesia; wwahyunawang@gmail.com (W.N.W.); ungkeajy@gmail.com (U.A.); abutholibaman@ugm.ac.id (A.T.A.); 3 Laboratory of Clinical Pathology, Abdul Wahab Sjahranie Hospital, Samarinda 75123, Indonesia; ayusriwahyunie@gmail.com; 4 Laboratory of Clinical Pathology, Tarakan Hospital, Jakarta10150, Indonesia; farasaad@yahoo.com; 5 Department of Clinical Pathology, Faculty of Medicine, Universitas GadjahMada/Sardjito Hospital, Yogyakarta 55281, Indonesia; adahesihdewi@yahoo.com; 6 Laboratory of Clinical Pathology, Prof. Dr. WZ Johannes Hospital, Kupang 85112, Indonesia; graciella1602@yahoo.com; 7 Department of Microbiology, Faculty of Medicine, Universitas Hasanuddin/Hasanuddin University Hospital, Makassar 90245, Indonesia; dr.munawirmuhammad@yahoo.co.id; 8 Department of Microbiology, Faculty of Medicine, Universitas Indonesia/Cipto Mangunkusumo Hospital, Jakarta 10430, Indonesia; delly.c.lestari@gmail.com; 9 Department of Clinical Pathology, Faculty of Medicine, Universitas Airlangga/Dr. Soetomo Hospital, Surabaya 60286, Indonesia; dr_aryati@yahoo.com; 10 Department of Clinical Pathology, Faculty of Medicine, Universitas Padjadjaran/Hasan Sadikin Hospital, Bandung 40161, Indonesia; idaparwati2008@gmail.com; 11 Department of Clinical Pathology, Faculty of Medicine, Universitas Indonesia/Cipto Mangunkusumo Hospital, Jakarta 10430, Indonesia; tonnyloho@yahoo.com; 12 Department of Clinical Pathology, Faculty of Medicine, Universitas Lambung Mangkurat/Ulin Hospital, Banjarmasin 70233, Indonesia; indahhariadi@gmail.com; 13 Laboratory of Clinical Pathology, Dr. Zainoel Abidin Hospital, Banda Aceh 24415, Indonesia; vividahril@yahoo.com; 14 Department of Clinical Pathology, Faculty of Medicine, Universitas Sumatera Utara/H. Adam Malik Hospital, North Sumatera 20136, Indonesia; loesnihari@yahoo.co.id; 15 Laboratory of Microbiology, Eka Hospital, Pekanbaru 28293, Indonesia; dewianggrainiyovi@gmail.com; 16 Department of Microbiology, Faculty of Medicine, Universitas Brawijaya/Saiful Anwar Hospital, Malang 65112, Indonesia; siwi_fk@ub.ac.id; 17 Lao-Oxford-Mahosot Hospital-Wellcome Trust Research Unit, Microbiology Laboratory, Mahosot Hospital, Vientiane, Laos; david.d@tropmedres.ac; 18 Centre for Tropical Medicine and Global Health, Nuffield Department of Clinical Medicine, Old Road Campus, University of Oxford, Oxford OX3 7FZ, UK; direk@tropmedres.ac; 19 Faculty of Infectious and Tropical Diseases, London School of Hygiene and Tropical Medicine, London WC1E 7HT, UK; 20 Tropical and Emerging Infectious Diseases Division, Menzies School of Health Research, Casuarina, Northern Territory 0811, Australia; bart.currie@menzies.edu.au; 21 Mahidol-Oxford Tropical Medicine Research Unit, Faculty of Tropical Medicine, Mahidol University, Bangkok 10400, Thailand; 22 Department of Microbiology, Faculty of Medicine, Universitas Gadjah Mada/Sardjito Hospital, Yogyakarta 55281, Indonesia; 23 Department of Microbiology, Faculty of Medicine, Universitas Udayana/Sanglah Hospital, Bali 80113, Indonesia; nyomansribudayanti@gmail.com; 24 Primate Research Center, Bogor Agricultural University, Bogor 16151, Indonesia; atie@indo.net.id

**Keywords:** *Burkholderia pseudomallei*, melioidosis, Indonesia

## Abstract

A recent modeling study estimated that there could be as many as 20,000 human melioidosis cases per year in Indonesia, with around 10,000 potential deaths annually. Nonetheless, the true burden of melioidosis in Indonesia is still unknown. The Indonesia Melioidosis Network was formed during the first melioidosis workshop in 2017. Here, we reviewed 101 melioidosis cases (99 human and two animal cases) previously reported and described an additional 45 human melioidosis cases. All 146 culture-confirmed cases were found in Sumatra (*n* = 15), Java (*n* = 104), Kalimantan (*n* = 15), Sulawesi (*n* = 11) and Nusa Tenggara (*n* = 1). Misidentification of *Burkholderia* *pseudomallei* was not uncommon, and most cases were only recently identified. We also evaluated clinical manifestations and outcome of recent culture-confirmed cases between 2012 and 2017 (*n* = 42). Overall, 15 (36%) cases were children (age <15 years) and 27 (64%) were adults (age ≥15 years). The overall mortality was 43% (18/42). We conducted a survey and found that 57% (327/548) of healthcare workers had never heard of melioidosis. In conclusion, melioidosis is endemic throughout Indonesia and associated with high mortality. We propose that top priorities are increasing awareness of melioidosis amongst all healthcare workers, increasing the use of bacterial culture, and ensuring accurate identification of *B. pseudomallei*and diagnosis of melioidosis.

## 1. Introduction 

Melioidosis in Indonesia was first diagnosed in Cikande, on Java island, in 1929 [1]. From then to 1960, a few additional cases were reported in Jakarta, Bogor and Surabaya, on Java island [2,3,4,5]. More recent reports concerned four culture-confirmed melioidosis cases among tsunami survivors in Banda Aceh, Sumatra, in 2005 [6], 51 culture-confirmed melioidosis patients in Malang, Java from 2011 to 2013 [7], and three culture-confirmed melioidosis patients in Makassar and Luwu Timur, Sulawesi, in 2013 [8]. Nonetheless, the reported cases are likely to be the tip of the iceberg and the true burden of melioidosis in Indonesia is still unclear.

A recent modeling study estimated the annual number of human melioidosis cases in Indonesia at 20,038, with 10,224 deaths annually if mortality was 51% [9]. This alarming estimate is possible, considering that, in Indonesia with a total population of about 260 million, about 1.6 million die every year, and about 350,000 and 150,000 of those who die are estimated to have communicable diseases and diabetes, respectively, defined as the primary causes of death using International Classification of Diseases (ICD) principles and Global Burden of Disease (GBD) analysis [10]. If melioidosis was an undiagnosed contributory cause in only 2% of these, this would account for 10,000 deaths [9]. The under-diagnosis and under-reporting of melioidosis worldwide are considered to be due to a lack of diagnostic microbiology laboratories serving the poor rural populations that are at greatest risk of infection, and a lack of awareness of the disease amongst physicians and laboratory staff [9,11]. Even good microbiological laboratories may initially miss the diagnosis and discard *B. pseudomallei* as a contaminant, especially in non-endemic areas [9,12]. Recent evidence suggests that, in Indonesia, where melioidosis is possibly highly endemic countrywide [9], capacity and utilization of bacterial cultures is limited [13], that misidentification of *B. pseudomallei* as another species or a contaminant is common, and that awareness of the disease among physicians and laboratory staff is very low [6,7,8]. 

Under a collaboration between Primate Research Center, Bogor Agricultural University and Health Security Partners (HSP), a workshop on ‘Melioidosis: Detection, Diagnosis, Treatment and Prevention Using a One-Health Approach’ was held in Bogor, Indonesia, from 14 to 16 August 2017. A total of four doctors, 12 veterinarians, seven microbiologists, one clinical pathologist and 16 other healthcare providers from 33 institutions attended, and the potential burden and challenges of melioidosis were discussed among participants. The Indonesia Melioidosis Network was formed during the meeting. In addition, the Indonesian Association of Clinical Microbiologists (PAMKI) held a session on ‘Epidemiology and Clinical Aspect of Melioidosis’ during their Annual Scientific Meeting in Padang, from 12 to 14 October 2017, and more evidence of melioidosis in Indonesia was additionally reported at this meeting. 

Here, we review the known and additional evidence of melioidosis in Indonesia, present the results of a surveillance study showing awareness and knowledge of the disease and the organism among healthcare providers in Indonesia, and discuss the needs and future challenges to save lives from melioidosis in Indonesia.

## 2. Melioidosis Cases and Presence of *B. pseudomallei* in Indonesia 

We performed (1) a retrospective review of published or reported melioidosis cases in Indonesia, (2) a retrospective study to identify unpublished culture-confirmed melioidosis cases in hospitals in which the microbiology laboratories had isolated *B. pseudomallei* from clinical specimens, and (3) a retrospective study to evaluate clinical manifestations of recent culture-confirmed patients from 2012 to 2017.

Firstly, we searched PubMed and SCOPUS for indigenous cases of melioidosis reported in Indonesia between 1 January 1921 and 30 November 2017, using the MeSH terms ‘melioidosis’ or ‘*pseudomallei*’. We also searched bibliographies from selected studies for secondary references. Our search included literature in English, Dutch, German and Indonesian.

**Figure 1:**
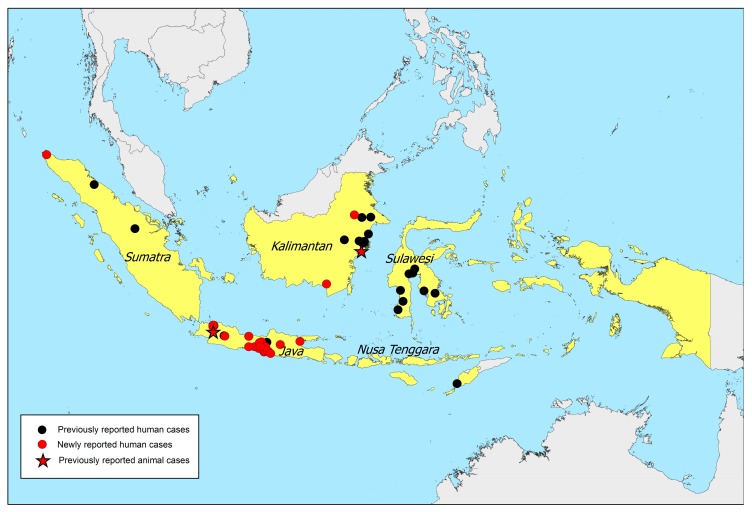
Location of 146 melioidosis cases. Black dots represent locations of 99 previously-reported human cases, red stars represent locations of two previously-reported animal cases and red dots represent locations of 45 newly-reported human cases. An interactive map is available at melioidosis.info website [25].

In addition to the 64 melioidosis cases previously reviewed [8], we found an additional 35 culture-confirmed human melioidosis cases and two animal cases (Table 1 and Figure 1). Prior to 1960, two additional cases were reported in Cimahi [14] and Salatiga [15] on Java island. We found that an additional report by Verbunt et al. in 1937 [16] was the same case described by Sudibyo [4]. All other cases were recently reported. The first animal case was reported in 2014, being a 3-year-old cynomolgus monkey (*Macaca fascicularis*) raised at Primate Research Center, Bogor [17]. At the 15th Asia-Pacific Congress on Clinical Microbiology and Infection in 2014, a human melioidosis case was reported from Medan, Sumatra [18]. At the first National Melioidosis Workshop in 2017, a human melioidosis case from Yogyakarta, Java [19], 13 cases from Abdul Wahab Sjahranie Hospital, Samarinda, East Kalimantan, eight cases from Dr. Wahidin Sudirohusodo Hospital, Makassar, South Sulawesi, and one case from Prof. Dr. WZ Johannes Hospital, Kupang, East Nusa Tenggara, were reported [20]. At the PAMKI meeting 2017, nine melioidosis cases presenting at Eka Hospital, Pekanbaru, Sumatra [21], and the second animal case from Samboja, Kalimantan [22] were reported. The animal was a Bornean orangutan (*Pongo pygmaeus*) of unknown age from the Borneo Orangutan Survival Foundation in Samboja, East Kalimantan. Both animal cases died shortly after clinical presentation, and the diagnosis was made post-mortem [17,22].

Secondly, to evaluate whether melioidosis had been diagnosed by routine microbiological laboratories in Indonesia but had not been reported, at the first National Melioidosis Workshop and at the PAMKI meeting, we requested participants to review the microbiology laboratory results in their hospitals to determine whether *B. pseudomallei* had been identified. To avoid duplication, cases that had already been reported (Table 1) were not included. 

We received information of an additional 45 melioidosis cases (Table 2). A total of 18 and five culture-confirmed melioidosis cases were observed at Sardjito Hospital, Yogyakarta, and at Tarakan Hospital, Jakarta, respectively, from 2012 to 2017. In addition*, B. pseudomallei* was isolated from clinical specimens at Dr. Zainoel Abidin Hospital, Banda Aceh (*n* = 1), Cipto Mangunkusumo Hospital, Jakarta (*n* = 4), a private laboratory (Granostic), Surabaya (*n* = 8), Ulin Hospital, Banjarmasin (*n* = 1) and Hasan Sadikin Hospital, Bandung (*n* = 8). 

Figure 1 shows the locations of all 146 cases (144 human cases and two animal cases) noted in this article (Table 1 and Table 2). Locations were towns where the cases were living or likely to have acquired melioidosis, where such information was available. In the event that the patients’ home addresses were not available, the address of the hospitals where the patients presented were used.

Thirdly, we also requested permission to analyze anonymous information of patients diagnosed from 2012 to 2017 to describe clinical manifestations and outcome of recent cases of melioidosis in Indonesia (Table 3). The retrospective study was approved by the education and research departments of five participating hospitals. Overall, we obtained anonymous data of 45 human cases available from Yogyakarta (*n* = 18), Samarinda (*n* = 13), Makassar (*n* = 8), Jakarta (*n* = 5) and Kupang (*n* = 1). We excluded three cases with incomplete data. 

Of 42 patients included in the analysis, five (12%) were neonates (age <1 month). One and four neonatal cases were observed in Jakarta and Yogyakarta, respectively. All five neonatal cases were blood culture-positive for *B. pseudomallei*, and three of them died. Another seven cases were found in infants (age 1 month to <2 years) and three cases in children (age 2 to <15 years). Thus, of the total, 15 (36%) cases were children (age <15 years) and 27 (64%) were adults (age ≥15 years). Diabetes was the most commonly identified risk factor amongst adults (Table 3), 60% of the cases (25/42) were blood culture-positive for *B. pseudomallei*, and the overall mortality was 43% (18/42). 

Specimens, diagnostic methods, and antibiotic susceptibility results of those 42 cases with complete clinical data are described in Table 4. Of the 42 cases, only 15 (36%) had antibiotic susceptibility test (AST) results available. We were informed that AST was often not routinely performed for *B. pseudomallei* because the laboratory staff did not know the correct standard operating procedures, quality control and guidelines on how to handle and perform AST on *B. pseudomallei*. As some AST results were not typical for *B. pseudomallei,* including those previously reported from Malang [7], we note that the AST results may be inaccurate or some isolates might not actually be *B. pseudomallei*. As isolates are not routinely kept in Indonesia, a retrospective study for further evaluation is not possible. 

Nonetheless, a total of 10 isolates (five from Samarinda and five from Makassar) reported as *B. pseudomallei* were sent to the Indonesia Research Partnership on Infectious Disease (INA-RESPOND) reference laboratory at Tangerang Regional General Hospital for further characterisation. In addition, 16S rRNA gene sequencing [23] and a PCR assay targeting the type III secretion system of *B. pseudomallei* [24] were performed. Nine of ten isolates were confirmed as *B. pseudomallei* by PCR. One isolate from Makassar was later identified as *Burkholderia stabilis* using 16s sequence analysis. The patient was a 77 year-old male presenting with an acute ischaemic stroke. The positive blood culture was obtained 19 days after hospital admission due to a lack of clinical improvement of alteration of consciousness, and the patient had no other signs and symptoms of sepsis, suggesting that *B. stabilis* was probably a contaminant. This case was not included in the list of melioidosis cases described above. This also supports our hypothesis that some isolates reported as *B. pseudomallei* might be other bacteria, and that training for *B. pseudomallei* identification is critically needed, country-wide. 

Details of these nine confirmed isolates and associated clinical manifestations are described in Table 5. Further studies on the nine isolates, including AST, multilocus-sequence typing and whole genome sequence typing, are in progress. 

## 3. Current Recommendations and Availability of Measures against Melioidosis

Currently, there are no national guidelines for diagnosis, treatment and prevention of melioidosis in Indonesia. In response to the evidence of the emergence of melioidosis in Indonesia, the international consensus guidelines for diagnosis [11] and treatment [26] of melioidosis have been recommended by the Indonesian Melioidosis Network. 

At the first melioidosis workshop, it was recommended that the top priority was to provide education about melioidosis to all clinicians including general practitioners, internists, paediatricians, surgeons, and neurologists, at the hospitals where melioidosis cases have been found (Table 1 and Table 2). This could be done by clinical pathologists or microbiologists at case reviews or local meetings in each hospital. Clinicians should be advised to consider melioidosis in any patients presenting with a fever, and communication between clinicians and laboratory staff is recommended if melioidosis is highly suspected; for example, diabetic patients presenting with community-acquired sepsis. Abdul Wahab Sjahranie Hospital, Samarinda has already introduced these steps since September 2016, and the clinicians’ response has been positive, with communication between clinicians and laboratory staff occurring for suspected cases and for specimens collected. Two melioidosis cases were diagnosed in 2017 after the implementation of the measures described above. It is important for melioidosis to be included in the curriculum of all medical schools in Indonesia in the future as meliodosis is not currently included in the curricula of any of the medical schools in the country.

Secondly, a simple and easy-to-perform laboratory algorithm for the identification of *B. pseudomallei* from clinical samples, such as that described by Trinh et al. [27], should be implemented in all microbiological laboratories in Indonesia. Future plans include the delivery of a workshop on how to prepare Ashdown agar, identification of *B. pseudomallei*, antibiotic susceptibility testing, and biosafety issues at a national meeting for laboratory staff. Again, in the longer term, these should be included routinely in training programmes for laboratory technicians. Although Vitek 2 is the most common method used for identification of Gram-negative bacilli in Indonesia, there have been some problems with its use for the identification of *B. pseudomallei*, which may have led to under-reporting [28,29,30]. The BD Phoenix is also used for identification of isolates in some large hospitals in Indonesia. However, *B. pseudomallei* is not in the Phoenix database [31], and misidentification of all *B. pseudomallei* isolates as *B. cepacia* is probably occurring in those institutions [31]. These issues will need to be urgently addressed. Microbiology laboratories in Indonesia that need confirmatory tests for isolates suspected as *B. pseudomallei*, can send them to the INA-RESPOND reference laboratory for confirmation by PCR.

## 4. Surveillance Systems and Reporting of Melioidosis in Indonesia 

Melioidosis is not currently a notifiable disease in Indonesia. We are initially considering the establishment of an online system to enable reporting of melioidosis cases from all microbiology hospital laboratories in Indonesia, particularly from those in the Indonesia Melioidosis Network. The system could be similar to ProMED-mail [32] or that of the International Melioidosis Society [25] but specific to Indonesia. The other option would be to use either of these currently running systems. The appropriate surveillance system will be discussed further at the next meeting of the Indonesia Melioidosis Network. 

The aim of reporting is to understand the distribution, morbidity and mortality of culture-confirmed melioidosis cases in Indonesia. We are certain that the 146 culture-confirmed melioidosis cases observed to date are just the tip of the iceberg, and continuing and enhancing the reporting system will provide a better understanding of the true burden and distribution of the disease. The results of this surveillance system should be used to encourage health policy makers and the infectious diseases network in Indonesia to give melioidosis the priority it deserves.

## 5. Awareness of Melioidosis in Indonesia 

A questionnaire was developed to evaluate medical practitioners’ knowledge and awareness of melioidosis. From 21 August to 4 October 2017, an online questionnaire using Google forms was distributed by the WhatsApp application to multiple formal and informal networks of medical researchers in Indonesia. To reduce response bias, we embedded questions about awareness of melioidosis amongst those on other infectious diseases, including dengue and typhoid. Approval for the study was obtained from the Faculty Medicine Hasanuddin University Ethics Committee, Makassar, Indonesia. 

A total of 568 participants completed the questionnaire. The median age of participants was 33 years (IQR 29–36; range 20–65 years), and 196 (34%) were male. Their occupations were general practitioner (*n* = 373, 66%), clinical pathologist (*n* = 75, 13%), internist (*n* = 45, 8%), pulmonologist (*n* = 14, 2%), paediatrician (*n* = 9, 2%), obstetrician (*n* = 9, 2%), neurologist (*n* = 8, 1%), ophthalmologist (*n* = 8, 1%), anaesthesiologist (*n* = 5, 1%), surgeon (*n* = 5, 1%), cardiologist, (*n* = 4, 1%) and others (*n* = 13, 2%). Participants were from Sulawesi (*n* = 231, 41%), Java (*n* = 213, 38%), Kalimantan (*n* = 50, 9%), Sumatra (*n* = 28, 5%), Maluku (*n* = 16, 3%), Papua (*n* = 16, 3%), Nusa Tenggara (*n* = 7, 1%) and Bali (*n* = 6, 1%). 

A total of 323 (57%) participants reported that they had never heard of melioidosis, while all participants reported that they had heard of dengue and typhoid. Only 44% of participants (*n* = 249) accurately answered that melioidosis was caused by a bacterium, while 184, 21 and 114 answered that melioidosis was caused by a parasite, a virus and ‘I don’t know’, respectively. 98% (*n* = 555) and 95% (*n* = 539) accurately answered that dengue and typhoid were caused by a virus and a bacterium, respectively. Only 153 participants (27%) accurately answered that the recommended diagnostic tests for melioidosis included blood or urine culture (*n* = 151 and 60, respectively), 285 (50%) answered ‘I don’t know’ how to diagnose melioidosis, and the remaining participants answered inaccurately that the recommended diagnostic tests for melioidosis were stool exam (*n* = 19, 3%) or did not include bacterial culture (*n* = 111, 20%). Only 101 participants (18%) accurately answered that the recommended treatment for melioidosis included ceftazidime (*n* = 101), 231 (41%) answered ‘I don’t know’ how to treat melioidosis, and the remaining participants answered inaccurately that the recommended treatments for melioidosis were chloramphenicol (*n* = 56, 10%), fluoroquinolones (*n* = 14, 2%), antivirals (*n* = 24, 4%), supportive treatment without appropriate antibiotics (*n* = 137, 24%), or fluid management without appropriate antibiotics (*n* = 5, 1%). Eighty-seven percent of the participants stated that MoH should promote education about melioidosis to healthcare workers (*n* = 492) and that MoH should promote awareness of melioidosis to lay people (*n* = 495).

The results of this online questionnaire shows that knowledge about melioidosis is limited among healthcare workers in Indonesia. Education about melioidosis for medical students should be initiated immediately countrywide, and melioidosis should be included in continuing medical education in Indonesia. 

Limitations of this online questionnaire include the uneven distribution of participants, and that awareness and knowledge might be underestimated by the biased sampling or overestimated because the study was conducted after the first melioidosis workshop. 

## 6. Current and Future Challenges

We consider that increasing awareness of melioidosis amongst all healthcare workers is the top priority; however, support from all stakeholders is needed. Awareness amongst veterinary professionals also needs to be improved as infections with *B. pseudomallei* have been identified in domestic animals and also certain wildlife including non-human primates (NHP) [33,34,35,36,37].

The second challenge is to increase the use of bacterial culture in Indonesia. In this respect, the under-diagnosis of melioidosis is just a reflection of a generalized limited capacity and under-utilization of diagnostic microbiology in the country. Diagnostic microbiology services in Indonesia face multiple challenges, including: (1) the size and configuration of the Indonesian archipelago, which makes the provision of equitable microbiology services to all parts of the country difficult; (2) the limited number of trained laboratory staff relative to the total population of Indonesia; and (3) historical limitations in the financial and regulatory support from government to develop microbiology services. To overcome these problems, the Indonesian government has established a new national regulation, which includes the provision of microbiology services as one of the requirements for hospital accreditation. This new national regulation has been being implemented gradually in all government hospitals since 2018. We hope that, over the next few years, Indonesia will be able to increase its microbiological capacity considerably. 

To improve diagnosis of melioidosis, not only does the capacity of clinical microbiology laboratories need to be expanded [13], but all healthcare workers should also be informed about the importance of bacterial culture in patients presenting with sepsis [38]. Recent evidence suggests that bacterial culture is under-utilized in Indonesia compared to the country’s health expenditure, and this could be related to the reimbursement system for bacterial culture, local customs and practice of clinicians, and a lack of support from related stakeholders and organizations [13]. 

The third challenge is biosafety and biosecurity, as *B. pseudomallei* is classified as a Tier 1 (top tier) Select Agent in the United States that can affect both humans and animals and possibly cause occupational infections [39]. Biosafety guidance for laboratories is needed. All laboratories where melioidosis cases have been found should be evaluated for their facilities, safe practices, and biosecurity with additional training and resources provided if necessary.

Drug availability is not a challenge in Indonesia. Ceftazidime and carbapenems are widely available throughout the country, and may be used and reimbursed within Indonesia’s universal health system if the diagnosis can be made.

Future challenges, after diagnosis of melioidosis is improved and if the burden of melioidosis is shown to be as high as expected [9], are to prevent melioidosis by reducing exposure, for example by wearing protective gear such as rubber boots and gloves during exposure to soil. Preventive measures are most important for people with the following conditions: diabetes, heavy alcohol consumption, kidney disease, lung disease, cancer, receiving immunosuppressive therapy and cuts or sores on the skin. Indonesia is an agricultural country, in which the majority of the population are rice farmers, and more than 10 million people are diabetics. It is known that changing behaviour is complex, and a multifaceted intervention is required. In Thailand, there are numerous barriers to adoption of behaviours recommended for melioidosis prevention [40]. Developing an effective prevention programme to reduce people’s exposure to *B. pseudomallei* in the environment and to educate them to seek medical attention if melioidosis is suspected, will be a formidable challenge in an Indonesian context.

## Figures and Tables

**Table 1:** Previously reported indigenous human and animal melioidosis cases in Indonesia (*n* = 101 cases).

Year Presented (References)	Locations	Age(Years)/Gender, Nationality	Clinical Characteristics	Diagnostic Method	Outcome
1929 [1]	Cikande, Java	50/M, Indonesian	Chronic painless nodules in the left thigh	Culture of pus (biochemistry, phenotypic tests and virulence in animal model)	Died
1934 [2]	Jakarta, Java	38/M, Indonesian	Severe sepsis with pulmonary, splenic and prostatic abscesses	Culture of pus (biochemistry, phenotypic tests and virulence in animal model)	Died
1935 [3]	Surabaya, Java	25/F, Indonesian	Abscess in the right gluteal region	Culture of pus (biochemistry, phenotypic tests and virulence in animal model)	Fully recovered
1936 [4,16]	Bogor, Java	60/M, Indonesian	Skin lesion with ulcers on right lower leg after trauma	Culture of pus (biochemistry and phenotypic tests)	Fully recovered
1937 [4]	Jakarta, Java	55/M, Indonesian	Abscess left foot, originated from minor trauma while farming	Culture of pus (biochemistry and phenotypic tests)	Fully recovered
1938 [14] *	Cimahi, Java	48/Unknown, European	Pneumonia and splenic abscess	Culture of pus (biochemistry, phenotypic tests and virulence in animal model)	Died
1950 [5]	Surabaya, Java	28/F, European	Pain in the lower abdomen and high fever	Culture of abscess from the right ovary (biochemistry and phenotypic tests)	Fully recovered
1958 [15] *	Salatiga, Java	Unknown	Diarrhoea	Culture of stool (biochemistry and phenotypic tests)	Died
2005 [6]	Banda Aceh, Sumatra	4 patients; 15/F, 18 mo/M, 10/F and 13/F	Pneumonia	Culture of sputum (API20NE)	Fully recovered (*n* = 1) or reported as improving (*n* = 3)
2011–2013 [7]	Malang, Java	51 patients (unknown age and sex)	Unknown	Culture of sputum, blood, pus and urine (VITEK2)	Unknown
2012 [17] *	Bogor, Java	3/Unknown, cynomolgus monkey	General weakness, decreased appetite, dehydration and cough	Culture of pus (VITEK2)	Died
2013–2014 [8]	Luwu Timur (*n* = 1) and Makassar (*n* = 2), Sulawesi	3 patients; 41/M, 45/F and 26/M, Indonesian	Sepsis (*n* = 1), neck abscess, sepsis and pneumonia (*n* = 1), and abscess behind the left ear lobe (*n* = 1)	Culture of blood (*n* = 1) and pus (*n* = 2) (VITEK2)	Died (*n* = 2) or lost to follow-up (*n* = 1)
2013 [19] *	Yogyakarta, Java	53/F, Indonesian	Neck abscess, pain and dyspnoea.	Culture of pus (Microbact)	Fully recovered
2014 [18] *	Medan, Sumatra	13/M, Indonesian	Fever, dry cough, weight loss and abdominal abscesses	Culture of pus (VITEK2)	Fully recovered
2017 [22] *	Samboja, Kalimantan	Unknown age and sex, Borneo orangutan	Loss of appetite, malaise, less active and apparent fever.	Culture of lung, spleen, and livertissue (VITEK2)	Died
2010–2017 [21] *	Pekanbaru, Sumatra	9 patients (mean age 52 years; range 34–67 years), all males and all Indonesian	Pneumonia, sepsis, abscess, cellulitis, osteomyelitis, pericarditis, seizure and decreased consciousness, and chronic suppurative otitis media with intratemporal complication.	Culture of sputum (*n* = 4), blood (*n* = 3) and pus (*n* = 3) (VITEK2)	Unknown
2014–2017 [20] *	Samarinda, Kalimantan (*n* = 13), Makassar, Sulawesi (n = 8) and Kupang, Nusa Tenggara (*n* = 1)	22 patients (median age 53.5 years; range 4–69 years), 15 males and 7 females, and all Indonesian	Sepsis, pneumonia, alteration of consciousness, and localized abscesses	Culture of blood (*n* = 11), pus (*n* = 7), tissue (n = 2) and urine (*n* = 2) (VITEK2) (PCR assay targeting type III secretion system in 9 cases)	Died (*n* = 9), Fully recovered (*n* = 9), and Unknown (*n =* 4)

* Not included in the recent review of melioidosis in Indonesia, published in 2015 [8].

**Table 2:** Newly reported indigenous melioidosis human cases in Indonesia (*n* = 45 cases).

Year Presented	Locations	Age(years)/Gender, Nationality	Clinical Characteristics	Diagnostic Method	Outcome
2010	Ulin Hospital, Banjarmasin, Kalimantan	Unknown/M	Unknown	Culture of blood (VITEK2)	Died
2010–2017	Private laboratory, Surabaya, Java	8 patients (unknown)	Unknown	Culture of sputum (*n* = 4), blood (*n* = 2), urine (*n* = 1) and nasopharyngeal swab (*n* = 1) (VITEK2)	Unknown
2012–2016	Hasan Sadikin Hospital, Bandung, Java	8 patients (unknown)	Unknown	Culture of blood (*n* = 5), body fluid (*n* = 3; unknown type of body fluid), pus (*n* = 1) (VITEK2)	Unknown
2012–2017	Cipto Mangunkusumo Hospital, Jakarta, Java	4 patients (unknown)	Unknown	Culture of blood (*n* = 1), pus (*n* = 1), sputum (*n* = 1), cerebrospinal fluid (*n* = 1) (VITEK2)	Unknown
2012–2017	Tarakan Hospital, Jakarta, Java	5 patients, 1 mo/M, 3 mo/M, 10 do/M, 2 mo/M and 59/M	Pneumonia (2), diarrhoea (1), alteration of consciousness (2)	Culture of blood (*n* = 4) and sputum (*n* = 1) (Microgen)	Died (*n* = 2), Fully recovered (*n* = 3)
2012–2017	Sardjito Hospital, Yogyakarta, Java	18 patients (median age 7.5 years; range 1 day–78 years), 13 males and 5 females, and all Indonesian	Sepsis, pneumonia, alteration of conscious, localized abscesses and urinary tract infection.	Culture of blood (*n* = 11), pus (n = 3), and urine (*n* = 5) (VITEK2)	Died (*n* = 7), Fully recovered (*n* = 11)
2017	Zainoel Abidin Hospital, Banda Aceh, Sumatra	33/M	Unknown	Culture of endotracheal secretion (Vitek2)	Unknown

**Table 3:** Demographic data, clinical presentations, risk factors and outcomes of 42 culture-confirmed melioidosis cases with available clinical data from 2012 to 2017.

Characteristics	Total Patients (*n* = 42)	Pediatric Patients (*n* = 15)	Adult Patients (*n* = 27)
Demographic information			
Median age (IQR and range)	41.5y (8.8m–56y, 1d–78y	2m (10d–9.5m, 1d–11y)	55y (47–59.5y, 21–78y)
Male sex	32 (76%)	10 (67%)	22 (82%)
Organ involvement *			
Bacteraemia	25 (60%)	14 (93%)	11 (41%)
Pneumonia	11 (25%)	3 (20%)	8 (30%)
Skin and Soft tissue	9 (21%)	1 (7%)	8 (30%)
Genitourinary	7 (17%)	0 (0%)	7 (26%)
Osteomyelitis	1 (3%)	0 (0%)	1 (4%)
Neurological	1 (3%)	0 (0%)	1 (4%)
Known risk factors **			
Diabetes mellitus	15 (36%)	0 (0%)	15 (56%)
Chronic kidney disease	5 (12%)	0 (0%)	5 (19%)
Chronic liver disease	2 (5%)	0 (0%)	2 (7%)
Malignancy	2 (5%)	0 (0%)	2 (7%)
Alcohol abuse	1 (2%)	0 (0%)	1 (4%)
Chronic lung disease	1 (2%)	0 (0%)	1 (4%)
Malnutrition	1 (2%)	1 (7%)	0 (0%)
None known	21 (50%)	14 (93%)	7 (26%)
Outcomes			
Full recovery	23 (55%)	8 (53%)	15 (56%)
Died	18 (43%)	7 (47%)	11 (41%)
Unknown	1 (2%)	0 (0%)	1 (4%)

* Bacteraemia was defined as blood culture positive for *B. pseudomallei*. Pneumonia was defined as a clinical diagnosis of pneumonia made by attending physicians (*n* = 7), having productive cough at clinical presentation (*n* = 5) or sputum culture positive for *B. pseudomallei* (*n* = 1). Skin and soft tissue involvement was defined as infections of non-skeletal tissue surrounding or supporting organs and other structures including subcutaneous tissue, muscle and lymph nodes (*n* = 9) or pus culture positive for *B. pseudomallei* (*n* = 8). Genitourinary involvement was defined as urine culture positive for *B. pseudomallei* (*n* = 7)*.* Osteomyelitis was defined as infection of bone (*n* = 1) or pus from bone culture positive for *B. pseudomallei* (*n* = 1); Neurological involvement was defined in a case presenting with sepsis and left hemiplegia. ** Risk factors were defined based on diagnoses made by attending physicians. Six adult patients had two known risk factors. IQR: Interquartile range.

**Table 4:** Specimens, diagnostic method and reported but unverified antibiotic susceptibility test results in 42 culture-confirmed melioidosis cases with available clinical data from 2012 to 2017.

Characteristics	Total (*n* = 42)
Specimens *	
Blood	25 (60%)
Pus	8 (19%)
Urine	7 (17%)
Tissue **	2 (5%)
Sputum	1 (2%)
Diagnostic method	
Vitek 2 identification system	37 (88%)
Microgen	5 (12%)
Antibiotic susceptibility test	
Not done	27 (64%)
Done ***	
Gentamicin (S)	0/13 (0%)
Amoxicillin-clavulanic acid (S)	2/5 (40%)
Ceftazidime (S)	12/14 (86%)
Doxycycline (S)	7/9 (78%)
Meropenem (S)	14/15 (93%)
Imipenem (S)	2/2 (100%)
Trimethoprim-sulfamethoxazole (S)	6/7 (86%)

* One adult patient had two culture-positive specimens. ** Tibial tissue (1), scrotal tissue (1). *** Data are number of isolates demonstrating susceptibility to the antimicrobial over the total number of isolates tested (%). Data are from the microbiology laboratories that had isolated *B. pseudomallei* from clinical specimens. Some AST results were not typical for *B. pseudomallei,* including resistance to amoxicillin-clavulanic acid, ceftazidime, doxycycline, meropenem and trimethoprim-sulfamethoxazole. We note that the AST results may be inaccurate or some isolates might not actually be *B. pseudomallei*.

**Table 5:** Demographics of nine patients with *B. pseudomallei* confirmed with PCR assays *.

Bacterial Strain	Year of Isolation/ Location of Isolation	Strain Source/Clinical Manifestations	Outcome
HBPMS00001	2015/Konawe, Southeast Sulawesi	Tibial tissue of 55-year old male patient presenting with open wounds with purulent discharge from legs, cough and fatigue	Fully recovered
HBPSK00002	2016/Samarinda, East Kalimantan	Pus of 55-year-old female patient with unknown clinical characteristics	Unknown
HBPMS00003	2016/Kolaka, Southeast Sulawesi	Blood of 56-year-old female patient presenting with decreased consciousness, generalized seizure, focal seizure of hand, headache, fever, swollen knee.	Died
HBPMS00004	2016/Luwu Utara, South Sulawesi	Pus of 39-year-old female patient presenting with lump on neck and weight loss.	Fully recovered
HBPMS00005	2016/Pinrang, South Sulawesi	Blood of 53-year-old male patient presenting with decreased consciuousness, fever, productive cough, shortness of breath, nausea, vomiting, abdominal pain and bloating. Icteric sclera and skin. Left leg swollen, pain and tenderness.	Died
HBPSK00001	2016/Kutai Timur, East Kalimantan	Blood of 4-year-old female patient presenting with fever, petechiae, poor appetite, anaemia	Died
HBPSK00003	2016/Kutai Timur, East Kalimantan	Pus of 37-year-old female patient presenting with skin ulcer on neck, fever	Fully recovered
HBPSK00004	2017/Kutai Kartanegara, East Kalimantan	Blood of 61-year-old male patient presenting with right hemiplegia, fever, decreased consciousness.	Died
HBPSK00005	2016/Samarinda, East Kalimantan	Urine of 44-year-old male patient presenting with fever, abscess on knee	Fully recovered

* 16S rRNA gene sequencing [23] and a PCR assay targeting the type III secretion system of *B. pseudomallei* [24].
